# Intrinsic cerebellar functional connectivity of social cognition and theory of mind in first-episode psychosis patients

**DOI:** 10.1038/s41537-021-00193-w

**Published:** 2021-12-03

**Authors:** Soo Hwan Park, Taekwan Kim, Minji Ha, Sun-Young Moon, Silvia Kyungjin Lho, Minah Kim, Jun Soo Kwon

**Affiliations:** 1 Geisel School of Medicine at Dartmouth, Hanover, NH USA; 2 Department of Brain and Cognitive Sciences, Seoul National University College of Natural Sciences, Seoul, Republic of Korea; 3 Department of Neuropsychiatry, Seoul National University Hospital, Seoul, Republic of Korea; 4 Department of Psychiatry, Seoul National University College of Medicine, Seoul, Republic of Korea; 5 Institute of Human Behavioral Medicine, SNU-MRC, Seoul, Republic of Korea

**Keywords:** Schizophrenia, Psychosis, Neural circuits, Biomarkers

## Abstract

Neuroimaging studies have revealed how intrinsic dysconnectivity among cortical regions of the mentalizing network (MENT) and the mirror neuron system (MNS) could explain the theory of mind (ToM) deficit in schizophrenia patients. However, despite the concurrent involvement of the cerebellum with the cortex in social cognition, the dysfunction in intrinsic interplay between the cerebellar nodes of MENT/MNS and the cortex in schizophrenia patients remains unknown. Thus, we aimed to investigate whether resting-state cerebello–cortical dysconnectivity exists in first-episode psychosis (FEP) patients in relationship with their ToM deficit. A total of 37 FEP patients and 80 healthy controls (HCs) underwent resting-state functional magnetic resonance imaging. Using a priori-defined cerebellar seeds that functionally connect to the MENT (right crus II) and MNS (right crus I), we compared cerebello–cortical functional connectivities (FCs) in FEP patients and HCs. Correlations between cerebello–parietal connectivities and ToM performance were investigated in FEP patients. FEP patients showed hyperconnectivity between the right crus II and anterior cingulate gyrus and between the right crus I and supplementary motor area, bilateral postcentral gyrus, and right central/parietal operculum (CO/PO). Hypoconnectivity was found between the right crus II and left supramarginal gyrus (SMG) in FEP patients. FCs between the right crus II and left SMG and between the right crus I and right CO/PO were significantly correlated with ToM scores in FEP patients. In accordance with the “cognitive dysmetria” hypothesis, our results highlight the importance of cerbello-cortical dysconnectivities in understanding social cognitive deficits in schizophrenia patients.

## Introduction

Patients with schizophrenia are consistently reported to have a deficit in theory of mind (ToM), the ability to represent the mental lives of others^1^. This social cognitive deficit can strongly predict community functioning; hence, elucidating the biological mechanism of the ToM deficit is likely to lead to the development of targeted treatments and improvements in social outcomes^1^. One theory that may explain this deficit is “cognitive dysmetria”, which views the diverse symptoms of schizophrenia as arising from a disrupted cortico-cerebellar-thalamic-cortical circuit (CCTCC), particularly the error detection function of the cerebellum^2,3^. Although a growing number of task-based functional magnetic resonance imaging (task-fMRI) studies and resting-state fMRI (rs-fMRI) studies have explained many of the psychotic symptoms of schizophrenia in terms of altered activity in the cerebellum and cerebellar functional connectivity (FC)^2,4–7^, there is no consensus on the link between cerebello–cortical FC abnormalities and ToM impairment in schizophrenia patients.

Despite the accumulating theoretical importance of the social cerebellum^8^, previous neuroimaging studies investigating ToM impairment in schizophrenia patients have held a corticocentric view^9,10^. Task-fMRI has revealed abnormally activated cortical regions in schizophrenia patients, such as the temporoparietal junction, premotor, and parietal areas, which largely belong to either the mentalizing network (MENT) or the mirror neuron system (MNS)^9,10^, two networks that are believed to mediate one’s ability to think about others’ internal states by the respective generation of a high-level reflective process and a low-level prereflective process^11–14^. rs-fMRI scans revealed dysconnectivity among the cerebral nodes of the MENT and MNS in patients with schizophrenia and patients with first-episode psychosis (FEP) and how these disrupted connectivities could explain patients’ symptom severity^15^ and impaired ToM task performance^16^. However, the MNS and MENT also extend to the cerebellum^8^, and there is evidence of abnormal cerebellar activation during ToM tasks in patients with schizophrenia^7,17^. Hence, the results from rs-fMRI focusing on the FC of the social cerebellum may help establish a relationship between abnormal cerebellar connectivity patterns during rest and activation patterns during ToM tasks in schizophrenia patients, similar to the association between hyperactivation and hyperconnectivity of the default mode network (DMN) in patients^18^. However, considering the role of the cerebellum in detecting and correcting errors, the cerebellar connectivity patterns with cortical regions that were abnormally activated during ToM tasks may be different from, even perhaps opposite to, those observed within the DMN. Therefore, the intrinsic connections that the cerebellar nodes of the MNT/MENT make with the cortex in schizophrenia patients and their relationship with their ToM deficits must first be understood.

While previous studies have attempted to explain the social cognitive deficits present in people with various neuropsychiatric disorders with abnormal cerebellar FC, none have focused on ToM impairment in people with schizophrenia^19–21^. Notably, Stoodley et al.^19^ demonstrated that neuromodulation of the right crus I in neurotypical humans could cause altered FC with the inferior parietal lobule, the same circuit that shows abnormal FC in children with autism and abnormal structural connectivity of Purkinje neurons in an autism mouse model. Moreover, chemogenetically mediated inhibition of the right crus I resulted in social impairments in mice, which could in fact be rescued by stimulating the same area^19^. The significance of the circuit between the right crus I and II and the left parietal cortex in social cognition was further highlighted, as it was shown to be the strongest correlate of social cognitive performance in people with psychotic disorders and neurotypical adults^20^. However, the employed social cognitive test measures only emotion management and regulation and does not assess features of higher-order social reasoning, such as ToM^20^. While these studies raise the suspicion that cerebello–parietal dysconnectivity present in patients with various neurodevelopmental disorders may explain their social cognitive deficits, the specific FC patterns and their relationship with ToM impairment in schizophrenia are still unknown.

Here, we aim to provide evidence for “cognitive dysmetria” and emphasize the need to include the cerebellum when investigating the neural mechanism of ToM impairment in schizophrenia patients. A recent meta-analysis of neuroimaging studies on ToM has revealed five cerebellar coordinates: two pertaining to MENT (right crus II and left crus II) and three pertaining to MNS (right crus I and lobule VIII)^8^. We compared resting-state cerebello–cortical connectivities with these cerebellar MENT/MNS nodes as seed regions in patients with FEP and healthy controls (HCs), as studies on patients with FEP are less vulnerable to the effects of illness chronicity, medication exposure, and chronically deficient social function that are usually present in patients with long-term schizophrenia. The cerebellum receives error signals and sends correction signals back to the cortex; if the hyperactivation pattern of the cortical region during a task is also present during rest, there may be less need for communication (i.e., hypoconnectivity) between the cortical region and the cerebellum, and vice versa. Thus, we posited that the cerebello–cortical FC patterns would contrast with the hyperactivation-hyperconnectivity association observed in the DMN of schizophrenia patients^18^. Specifically, we hypothesized that FEP patients during rest would show hyperconnectivity with regions that showed hypoactivation during ToM tasks and hypoconnectivity with regions that showed hyperactivation during ToM tasks in patients with schizophrenia^9,10^. Based on past evidence demonstrating the role of the cerebello–parietal circuit in social impairment^19,20^, the cerebello–parietal FC were hypothesized to correlate with ToM impairment in patients with FEP.

## Results

### Subject characteristics and ToM scores

The sociodemographic and clinical characteristics, as well as the ToM task scores of the subjects, are summarized in Table 1. No significant differences in sociodemographic characteristics were observed between groups, except intelligence quotient (IQ), as the patients with FEP scored significantly lower than HCs (*t*_116_ = −5.413, *p* < 0.001). Of the 37 patients, 30 were medicated with atypical antipsychotics at the time of data acquisition.

**Table 1:** Sociodemographic, clinical, and theory of mind (ToM) task performance characteristics of subjects.

	FEP (*n* = 37)	HCs (*n* = 80)	Statistics (df)	*p* value
Age (years)	23.05 (5.64)	22.99 (4.76)	0.066 (116)	0.947
Sex (M/F)	16/21	48/32	2.867 (1)	0.090
IQ	98.49 (13.66)	112.43 (12.62)	−5.413 (116)	<0.001**
Handedness (R/L)	32/5	75/5	1.708 (1)	0.191
PANSS total	67.73 (12.58)			
Positive symptoms	16.32 (4.49)			
Negative symptoms	16.97 (4.84)			
General symptoms	34.43 (6.83)			
Medication (medicated/drug-naïve; average olanzapine-equivalent dose in mg/day)	30/7 (11.64)			
False belief total score	7.70 (2.26)	9.70 (2.16)	8.604 (1,113)	0.004*
First-order false belief	4.46 (1.38)	5.05 (1.24)	2.308 (1,113)	0.131
Second-order false belief	3.24 (1.59)	4.65 (1.54)	8.048 (1,113)	0.005*

The sociodemographic group difference in continuous variables was tested using the independent samples *t* test (t-statistic is reported), categorical variables with the chi-square test (*χ*^2^ is reported), and ToM scores with one-way analysis of covariance with intelligence quotient (IQ) as the covariate (*F*-statistic is reported).

*FEP* first-episode psychosis, *HC* healthy control, *PANSS* Positive and Negative Syndrome Scale.

*p < 0.01; **p < 0.001.

Univariate statistics controlling for IQ revealed significant group differences in ToM task scores; FEP patients performed significantly worse than HCs in the false belief task (*F*_1,113_ = 8.604, *p* = 0.004). Further analysis of false belief subscores revealed that FEP patients scored significantly lower than HCs in second-order (*F*_1,113_ = 8.048, *p* = 0.005), but not first-order (*F*_1,113_ = 2.308, *p* = 0.131), false belief tasks. Second-order false belief performance correlated significantly with IQ in our participants (*r* = 0.40, *p* < 0.001) but not with symptom severity or medication dosage in the patients. No significant correlation was observed between IQ, symptom severity, or medication dosage and first-order false belief performance.

### Seed-based resting-state FC

**Fig. 1:**
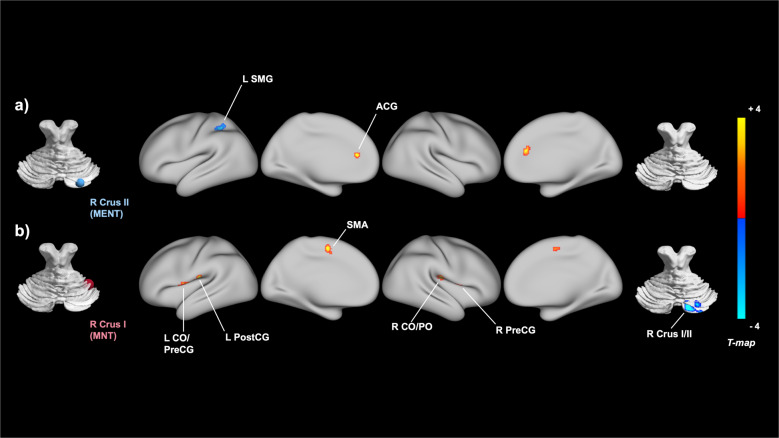
Brain regions showing between-group connectivity differences with social cerebellar regions of interest (ROIs) from seed-to-voxel analysis. Significant clusters of the between-group analyses obtained for the two cerebellar ROIs are presented in a combined view: **a** right crus II, which is functionally connected to the mentalizing network (MENT), and (**b**) right crus I, which is functionally connected to the mirror neuron system (MNS). Blue and red clusters indicate significant hypoconnectivity and hyperconnectivity with the cerebellar seeds, respectively. R right, L left, SMG supramarginal gyrus, ACG anterior cingulate gyrus, CO central operculum, PreCG precentral gyrus, PostCG postcentral gyrus, SMA supplementary motor area, PO parietal operculum; R Crus II (MNI coordinates +26, −84, −32); and R Crus I (+40, −48, −32). Statistical significance of the cluster threshold was set at false discovery rate (FDR)-corrected *p* < 0.05. The color bar indicates *T*-values.

For each of the five a priori-defined cerebellar seeds, we assessed and compared its whole-brain FC in patients with FEP and HCs. The analysis controlling for the effects of IQ revealed that FEP patients, compared to HCs, displayed hyperconnectivity between the right crus I and the supplementary motor area (SMA), the left central operculum/precentral gyrus, the right precentral gyrus, and the right central/parietal operculum (CO/PO). Compared to HCs, FEP patients also showed hypoconnectivity between the right crus I and the right crus II, a region heavily overlapping with the cerebellar MENT seed. In addition to these five clusters that survived Bonferroni correction, FEP patients, compared to HCs, showed hypoconnectivity between the right crus II and left supramarginal gyrus (SMG) and hyperconnectivity between the right crus I and left postcentral gyrus and between the right crus II and anterior cingulate gyrus (ACG) at a trend level. The detailed results on all eight clusters that showed cerebellar functional dysconnectivity are summarized in Table 2 and Fig. 1. All images were generated using Connectome Workbench^22^ and BrainNet Viewer (https://helab.bnu.edu.cn/brainnet-viewer)^23^. For the three remaining cerebellar seeds located at the left crus II and lobule VIII, no significant clusters of group differences were observed.

**Table 2:** Brain regions demonstrating significant cerebellar connectivity differences in patients with first-episode psychosis (FEP) versus healthy controls (HCs).

Cerebellar seed (MNI coordinates)	Region of altered connectivity	MNI coordinates of peak	Cluster size	Cluster size p-FDR
FEP > HCs				
R Crus II (+26−84−32)	Anterior cingulate gyrus Paracingulate gyrus	+02 +38 +20	76	0.041
R Crus I (+40−48−32)	Supplementary motor area	−02 +0 +54	168	0.001*
	L Central operculum L Precentral gyrus	−56 −02 +08	149	0.001*
	R Precentral gyrus	+58 +06 +04	114	0.004*
	R Central/Parietal operculum	+52 −18 +14	106	0.005*
	L Postcentral gyrus	−68 −18 +16	71	0.026
FEP < HCs				
R Crus II (+26−84−32)	L Supramarginal gyrus L Superior parietal lobule	−44 −48 +54	90	0.039
R Crus I (+40−48−32)	R Crus I/II	+24 −80 −34	193	<0.001*

*R* right, *L* left, *MNI* Montreal Neurological Institute, *FDR* false discovery rate.

*Bonferroni-corrected *p* < 0.01 (0.05/5).

### Relationship between cerebello–parietal connectivity and ToM measures

To examine the relationship between cerebello–parietal dysconnectivity and ToM, the connectivity strengths between the right crus II and left SMG and between the right crus I and right CO/PO were selected for correlation analysis within the FEP group. According to the Montreal Neurological Institute (MNI) structural atlas, both clusters are located in the parietal lobe; in particular, the connectivity of the parietal lobe with the cerebellum has previously been associated with social cognitive performance in patients with autism and psychosis^19,20^. Additionally, these two clusters have previously been identified to show abnormal activity during ToM tasks in schizophrenia patients^9,10^. Although the connectivity strength between the right crus II and left SMG did not survive seed-level Bonferroni correction, we employed it in our correlation analysis due to its peak position in the parietal cortex and its trend level hypoconnectivity in FEP patients compared to HCs (i.e., significant uncorrected cluster size *p*-FDR value).

**Fig. 2:**
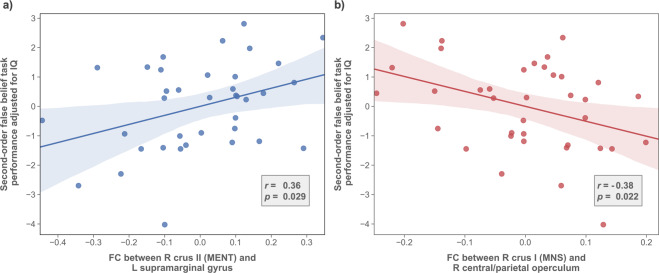
Relationship between cerebello–parietal functional connectivity (FC) and ToM performance in patients with first-episode psychosis (FEP). Scatter plots, regression lines, and their 95% confidence intervals for partial correlation analysis controlling for intelligence quotient (IQ) (**a**) between the right crus II and left SMG FC (*z* scores) and second-order false belief scores and (**b**) between the right crus I and CO/PO FC and second-order false belief scores in FEP patients are displayed. R right, L left, FC functional connectivity, MENT mentalizing network, MNS mirror neuron system.

As shown in Fig. 2, the analysis revealed a significant positive correlation between second-order false belief scores and right crus II and left SMG FC (*r* = 0.36, *p* = 0.029), as well as a significant negative correlation between second-order false belief scores and right crus I and right CO/PO FC (*r* = −0.38, *p* = 0.022).

## Discussion

This study investigated whether the patterns of resting-state cerebello–cortical FC in FEP patients are abnormal and examined whether cerebello–parietal dysconnectivity could explain the ToM deficits in these patients. From our seed-based intrinsic FC analysis, we observed a mixed pattern of cerebellar hyperconnectivity and hypoconnectivity in patients with FEP involving socially relevant regions. Specifically, compared with HCs, FEP patients displayed significant and trend-level hyperconnectivity between the right crus I and the SMA, left central operculum/precentral gyrus, left postcentral gyrus, right precentral gyrus, and right CO/PO, as well as between the right crus II and ACG. Moreover, compared with HCs, FEP patients showed significant and trend-level hypoconnectivity between the right crus II and left SMG and between the right crus I and right crus II. Additionally, second-order false belief scores were negatively correlated with right crus I and right CO/PO FC and positively correlated with right crus II and left SMG FC in patients. As the first study demonstrating the relationship between cerebello–parietal dysconnectivity and ToM deficits in FEP patients, our results emphasize the need to include the cerebellum in investigations of cognitive deficits in schizophrenia and support the “cognitive dysmetria” hypothesis by explaining a social cognitive deficit with disruptions in the CCTCC in the early stage of the disorder.

It should be noted that the cerebellar dysconnectivities in FEP patients revealed in our study largely involved the cortical regions (the SMA, left SMG, ACG, right CO/PO, and left pre- and postcentral gyri) of the MNS^8,12,24^. Consistent with previous research that demonstrated dysconnectivity within the MNS in patients with schizophrenia^15^ and between the MNS and MENT in patients with FEP^16^, our results suggested that the within- and between-network dysconnectivity patterns involving the cortical regions of the MNS extend to the social cerebellum. Interestingly, while these previous studies only noted significantly different hypoconnectivities in their patient samples, we observed a mixed pattern of cerebellar dysconnectivities in FEP patients. Specifically, we observed hypoconnectivity with regions that previously showed hyperactivation (left SMG) in schizophrenia patients when they were performing ToM tasks and hyperconnectivity with regions that showed hypoactivation (the ACG, right precentral gyrus, and right CO/PO) when schizophrenia patients performed such tasks^9^. This relationship is different from the abnormal activation-connectivity associations seen in the cortex and should be understood in terms of the functional role of the cerebellum. Previous research has demonstrated an association between hyperconnectivity of the DMN, which overlaps with MENT, and its abnormal hyperactivation in schizophrenia patients when these individuals are performing tasks^18^. Within the MNS, a similar association could be inferred in the other direction, as separate studies have shown hypoactivation during ToM tasks and intrinsic hypoconnectivity among such regions in patients^9,15^. However, this type of relationship may not translate to the cerebellum, which is highly regarded as the brain’s center for “error detection and correction”^25^. Specifically, the pattern of cerebello–cortical FC observed in patients may depend on the amount of error input from the cortex and the respective correction output from the cerebellum. It is plausible that patients’ cortical regions that have previously shown hypoactivation during ToM tasks were also hypoactivated during rest. Excessive communication between the cortex and cerebellum during rest may represent immoderate effort by the cerebellum to correct this deficiency. This pattern of FC is not new; schizophrenia patients have been frequently characterized by cerebello–cortical hyperconnectivity involving sensorimotor cerebral areas, which heavily overlap with the MNS^26,27^. The patients’ cerebellar hypoconnectivity can be similarly explained. These cortical regions may have been hyperactivated in patients during rest, as they were when patients were performing ToM tasks. Thus, cerebellar hypoconnectivity in patients may reflect an abnormally low need for error-related communication between the cerebellum and MNS. Further studies investigating the intrinsic cerebellar FC and the task activation patterns of the dysconnected cortical regions are needed, however, to determine the neural association.

Building on work that has established the involvement of the parietal cortex in social cognition^12,28^, the relationship between abnormal FC with the cerebellum and social cognitive deficits has been demonstrated by previous studies on autism and psychosis^19,20^. Both of these studies identified the connectivity between the left parietal cortex and right posterior cerebellar lobules as a predictor of social cognitive performance in their respective patient samples. As expected, our analysis revealed congruent results. The FC between the right crus II and left SMG, a key node in the MNS that aligns with the previously identified parietal regions, was positively correlated with ToM performance. Additionally, we observed a significant negative correlation between patients’ ToM performance and their FC between the right crus I and right CO/PO, another parietal region in the MNS in schizophrenia patients that was abnormally activated during ToM task performance. Our results suggest that the patients’ aberrant error-related signals from or to their cerebella may be associated with their poor social optimization and deficits in ToM. In addition, these correlations observed in the FEP patients in our study also align with recent studies that demonstrated associations between positive symptom severity and morphological differences in cerebellar gray and white matter across different clinical stages of schizophrenia^29,30^. Specifically, the significant correlation between bilateral crus I/II volume reductions in first-episode psychosis patients and the severity of their positive symptoms suggests how functional and structural alterations in the cerebellum may be a common link between positive symptom severity and ToM impairment in the early stages of schizophrenia. Overall, while our results extend the link between intrinsic cerebello–parietal dysconnectivity and social cognitive deficits to ToM impairment in FEP patients, the biological meaning of cerebellar dysconnectivity should still be cautiously interpreted, and special consideration should be given to the functional roles of the parietal regions.

Recently, the notion of predictive coding^31^ has led to the identification of the “prediction network”, which has been proposed to mediate mirroring, among other functions^32^. Surprisingly, this network, which includes the inferior frontal gyrus, SMA, ACG, parietal cortex, thalamus, and cerebellar crus I and II, heavily overlaps with the regions that showed cerebellar dysconnectivity in our study, suggesting that the dysconnectivity observed in the MNS may actually be the dysconnectivity within the functionally larger “prediction network”. This is in accordance with not only recent evidence demonstrating the role of cerebellar connectivity in social sequence prediction^33^ but also Andreasen’s parsimonious “cognitive dysmetria” theory of schizophrenia^3^, as the network overlaps with the major components of the CCTCC, as well as the novel predictive coding account of psychosis^34^. Furthermore, the significant correlations between abnormal intrinsic cerebellar FC and ToM performance, a measure of social prediction, in FEP patients suggest that some symptoms of schizophrenia may be a cognitive reflection of dysconnectivity in this larger network. Although our results add to the accumulating evidence that highlights the aberrant involvement of the cerebellum in predictive deficits of psychosis^35^, key gaps still remain to be filled that can elucidate how dysfunctions in the “prediction network” may manifest themselves as cognitive impairments of schizophrenia.

Our work has several limitations to note. First, because the regions of interest (ROIs) used in the study were derived from coordinates determined by the automated meta-analysis of NeuroSynth^8^, they may not represent the exact cerebellar locations that are functionally connected to the MNS or MENT in our sample. Because our study used spherical ROIs centered around the previously determined coordinates, the ROIs may also not conform to the precise shape of the cerebellar lobules. While we sought to minimize this error by referring to a meta-analysis of aberrant cerebellar activation patterns in patients with schizophrenia when they were performing ToM tasks^36^, the analysis covered only three studies. Hence, we believe that the cerebellar coordinates obtained from the NeuroSynth analysis were more reliable than other options. Nevertheless, it is possible that the time series obtained from our ROIs represent voxels outside of the socially relevant cerebellar regions. Second, although the false belief task used in our study has been widely used to measure ToM ability^37^, the subscores of our version have a narrow range^38,39^. Therefore, there is a greater chance that participants scored well beyond or below their actual ToM ability. Furthermore, because the false belief task used in our study may not accurately simulate social predictions in real-world settings that involve social optimization, future replications are encouraged to employ a more naturalistic ToM task that better identifies the participants’ social cognitive ability on the spectrum and takes the function of the cerebellum into account. Finally, the relatively long repetition time (TR) employed to obtain functional images may have led to a lower statistical power and larger time discrepancy within a single 3D volume compared with short-TR imaging^40^. Despite the statistical sacrifice stemming from long-TR imaging, whole-brain coverage was made possible, enabling us to study cerebellar connectivity. We aimed to minimize the within-volume temporal discrepancy through slice timing correction. Although the CCTCC is known to comprise polysynaptic closed loops, our work based on long-TR imaging mainly revolved around the correlation coefficient-based FC between the cerebellum and cortex and did not take directionality of connections into account, potentially limiting a deeper understanding of dysfunctions in error signals or the corresponding correction signals. Considering the growing evidence for dysconnectivity within the circuit as a characteristic pathophysiological aspect of schizophrenia^26,41^, future studies should consider employing an imaging sequence that allows a more fine-grained effective connectivity analysis when investigating the aberrant circuit and its association with cognitive deficits of the disorder.

Despite these limitations, our work establishes that aberrant cerebellar FC patterns are present in patients with FEP and suggests that cerebello–parietal dysconnectivity explains ToM deficits in patients. The results revealed in the study provide evidence for the “cognitive dysmetria” theory of schizophrenia and suggest that dysconnectivity within the MNS or its overlapping, domain-general “prediction network” may explain social cognitive deficits or other predictive deficits of schizophrenia. Future investigations of the relationship between cerebellar dysconnectivity and social cognitive deficits are encouraged to employ methods and tasks that align more with the anatomy and functional role of the cerebellum.

## Methods

### Participants

Forty FEP patients were recruited from the Seoul Youth Clinic^42^ of Seoul National University Hospital (SNUH) from June 2010 to August 2016. Using the Structured Clinical Interview for DSM, fourth edition (DSM-IV), Axis I (SCID-I), experienced psychiatrists interviewed all FEP individuals who met the following inclusion criteria: between the age of 15 to 40; diagnosis of schizophrenia, schizoaffective disorder, or schizophreniform disorder according to the DSM-IV criteria; and symptom presence for less than 2 years. A total of 110 HCs were recruited from internet advertisements, screened, and confirmed using the SCID Nonpatient Edition (SCID-NP). They were excluded when they had any past or current SCID-NP axis I diagnoses and first- to third-degree biological relations with psychotic disorders. The exclusion criteria for all participants were a lifetime history of neurological disorders, clinically severe head trauma, substance use disorder (except nicotine), and intellectual disability (IQ < 70). From these participants, we only included participants with ToM task scores (excluding 2 FEP patients and 21 HCs) and structural and functional MRI data with intact cerebella (excluding 1 FEP patient and 9 HCs). In total, 117 participants (FEP patients, *n* = 37; HCs, *n* = 80) were included in the current study. From the same cohort, data from 26 FEP patients and 26 HCs were used in our previously published study^16^, and 37 FEP patients and 40 HCs were used in another study^43^.

Written informed consent was obtained from all subjects after they were provided with a thorough explanation of the study procedure in the previous prospective cohort study (Institutional Review Board (IRB) no. H-1110-009-380). In the case of minors, their parents provided written informed consent while they provided written informed assent. The study was conducted in accordance with the Declaration of Helsinki and was approved by the IRB of the SNUH (IRB no. H-2104-223-1216).

### ToM false belief task

ToM was measured with the short form of the false belief task^16,38,39,44,45^, which comprises first- and second-order tasks, provided in the validated Korean version of the ToM battery^46^. The first-order task assessed the ability of the subject to recognize a character’s false belief about reality^39^, while the second-order task evaluated a character’s knowledge of the mental state of the other character^38^. Each included a picture with a short vignette, followed by a comprehension question and a justification question; the latter measured the subject’s ability to infer the character’s mental state. The maximum score for each false belief subtask was 6; thus, the total maximum score was 12.

### Image acquisition and preprocessing

All MR images were acquired with a Siemens 3T Trio scanner (Siemens, Erlangen, Germany) using a 12-channel head coil. For each participant, a high-resolution 3D T1-weighted image was obtained using a magnetization-prepared rapid gradient echo sequence (TR/echo time (TE) = 1670/1.89 ms, field of view (FOV) = 250 mm, flip angle (FA) = 9°, voxel size = 1.0 × 1.0 × 1.0 mm^3^, and sagittal slices = 208) for anatomical reference. Using an echo planar imaging sequence (TR/TE = 3500/30 ms, FOV = 240 mm, FA = 90°, voxel size = 1.9 × 1.9 × 3.5 mm^3^, 35 axial slices), rs-fMRI data were collected for 6 min and 58 s. The phase-encoding direction for all images was anterior to posterior. During rest, participants were instructed to keep their eyes closed and relax but to not fall asleep. To ensure that participants had not fallen asleep, they completed a questionnaire after the scan. Visual inspection of functional images was completed to exclude participants with more than one missing cerebellar slice.

Image preprocessing was performed via the CONN toolbox (version 19c) as implemented in MATLAB 2020b (http://www.nitrc.org/projects/conn)^47^. After discarding the first four volumes for each subject, the remaining 112 functional images were processed in a standard preprocessing pipeline: realignment and unwarping, slice timing and head motion correction, ARtifact detection Tools (ART)-based outlier detection, coregistration of the functional and structural images, normalization into MNI space, and smoothing (6-mm full-width at half-maximum). The signals from white matter, cerebrospinal fluid, motion realignment parameters, and their first derivatives were regressed out (aCompCor strategy^48^), followed by linear detrending and bandpass filtering (0.008–0.09 Hz).

### FC analysis

To study cerebellar FC with socially relevant cerebral regions, five social cerebellar ROIs were chosen according to the MNI coordinates revealed by the automated meta-analysis of NeuroSynth with the keywords “action” and “mirror” for the MNS seeds and “mentalizing” for the MENT seeds^8^. Spherical ROIs (5 mm) were created, centered around these coordinates. The centers of the two MENT cerebellar ROIs were located at the right crus II (+26, −84, −32) and its left mirror location (−26, −84, −32), while those of the three MNS cerebellar ROIs were at the right crus I (+40, −48, −32) and lobule VIII (+15, −75, −50), (+25, −60, −50).

For each seed, the signals of its voxels were averaged and correlated with signals of voxels in the rest of the brain using a first-level general linear model to obtain the connectivity strength^47^. The coefficients of Pearson’s bivariate correlation were subsequently converted into normally distributed *z* scores via Fisher *r*-to-*z* transformation.

### Statistical analysis

Data analysis was performed using Statistical Package for Social Sciences (IBM SPSS Version 22). Independent *t* tests and *χ*^2^ tests were used to examine the demographic and clinical characteristics between FEP patients and HCs. ToM performance between the two groups was examined with analysis of covariance with IQ as the covariate, as it was significantly lower for the FEP group and significantly correlated with second-order false belief scores.

We compared the connectivity strengths between patients with FEP and HCs using a second-level general linear model while controlling for the effects of IQ and defined clusters showing significant group differences determined via Gaussian random field theory parametric statistics (cluster threshold: *p*-FDR corrected < 0.05; voxel threshold: *p*-uncorrected < 0.001)^49^. As there were five cerebellar seeds, the seed-level Bonferroni procedure was used to account for multiple comparisons (Bonferroni-corrected *p* < 0.01 (0.05/5)).

To investigate the relationship between ToM deficits and cerebello–parietal FC, we conducted partial correlation analysis among FEP patients between FC involving parietal regions that have previously shown abnormal activity during ToM tasks and the ToM task subscores that showed significant impairment^50^. Because only IQ, but not symptom severity and medication dosage, was correlated with ToM performance, it was controlled when examining this relationship.

### Reporting summary

Further information on research design is available in the Nature Research Reporting Summary linked to this article.

## Supplementary information

Reporting Summary

## Data Availability

The data that support the results of this study are available from the corresponding author upon reasonable request. The data are not publicly available because they contain information that might compromise the privacy of the research participants.

## Code availability

The code for data processing and statistical analysis in this study is available from the corresponding author upon reasonable request.
