# Supplementary material for: Intrinsic cerebellar functional connectivity of social cognition and theory of mind in first-episode psychosis patients
Source: NPJ Schizophr. 2021 Dec 3;7:59. doi: 10.1038/s41537-021-00193-w (PMC8642425; doi:10.1038/s41537-021-00193-w)
Supplement: Supplementary file 1 — Reporting Summary [file 41537_2021_193_MOESM1_ESM.pdf]

nature portfolio

Corresponding author(s): Minah Kim

Last updated by author(s): Oct 4, 2021

Reporting Summary

Nature Portfolio wishes to improve the reproducibility of the work that we publish. This form provides structure for consistency and transparency in reporting. For further information on Nature Portfolio policies, see our Editorial Policies and the Editorial Policy Checklist.

Statistics

For all statistical analyses, confirm that the following items are present in the figure legend, table legend, main text, or Methods section.

n/a	Confirmed	
☐	☒	The exact sample size (n) for each experimental group/condition, given as a discrete number and unit of measurement
☐	☒	A statement on whether measurements were taken from distinct samples or whether the same sample was measured repeatedly
☐	☒	The statistical test(s) used AND whether they are one- or two-sided<br>*Only common tests should be described solely by name; describe more complex techniques in the Methods section.*
☐	☒	A description of all covariates tested
☐	☒	A description of any assumptions or corrections, such as tests of normality and adjustment for multiple comparisons
☐	☒	A full description of the statistical parameters including central tendency (e.g. means) or other basic estimates (e.g. regression coefficient) AND variation (e.g. standard deviation) or associated estimates of uncertainty (e.g. confidence intervals)
☐	☒	For null hypothesis testing, the test statistic (e.g. F, t, r) with confidence intervals, effect sizes, degrees of freedom and P value noted<br>*Give P values as exact values whenever suitable.*
☒	☐	For Bayesian analysis, information on the choice of priors and Markov chain Monte Carlo settings
☒	☐	For hierarchical and complex designs, identification of the appropriate level for tests and full reporting of outcomes
☐	☒	Estimates of effect sizes (e.g. Cohen's d, Pearson's r), indicating how they were calculated

Our web collection on statistics for biologists contains articles on many of the points above.

Software and code

Policy information about availability of computer code

Data collection	No software was used.
Data analysis	Image preprocessing was performed via the CONN toolbox (version 19c) as implemented in MATLAB 2020b (http://www.nitrc.org/projects/conn).

For manuscripts utilizing custom algorithms or software that are central to the research but not yet described in published literature, software must be made available to editors and reviewers. We strongly encourage code deposition in a community repository (e.g. GitHub). See the Nature Portfolio guidelines for submitting code & software for further information.

Data

Policy information about availability of data

All manuscripts must include a data availability statement. This statement should provide the following information, where applicable:

- Accession codes, unique identifiers, or web links for publicly available datasets
- A description of any restrictions on data availability
- For clinical datasets or third party data, please ensure that the statement adheres to our policy

The data that support the results of this study are available from the corresponding author upon reasonable request. The data are not publicly available because they contain information that might compromise the privacy of the research participants.

March 2021

Field-specific reporting

Please select the one below that is the best fit for your research. If you are not sure, read the appropriate sections before making your selection.

☐ Life sciences ☒ Behavioural & social sciences ☐ Ecological, evolutionary & environmental sciences

For a reference copy of the document with all sections, see nature.com/documents/nr-reporting-summary-flat.pdf

Behavioural & social sciences study design

All studies must disclose on these points even when the disclosure is negative.

Study description	quantitative cross-sectional study.
Research sample	Forty first-episode psychosis (FEP) patients were recruited from the Seoul Youth Clinic of Seoul National University Hospital (SNUH). Using the Structured Clinical Interview for DSM, fourth edition (DSM-IV), Axis I (SCID-I), experienced psychiatrists interviewed all FEP individuals who met the following inclusion criteria: between the age of 15 to 40; diagnosis of schizophrenia, schizoaffective disorder, or schizophreniform disorder according to the DSM-IV criteria; and symptom presence for less than 2 years. A total of 110 healthy controls (HCs) were recruited from internet advertisements, screened, and confirmed using the SCID Nonpatient Edition (SCID-NP).
Sampling strategy	From 40 FEP and 110 HC participants, we only included participants with ToM task scores (excluding 2 FEP patients and 21 HCs) and structural and functional MRI data with intact cerebella (excluding 1 FEP patient and 9 HCs). In total, 117 participants (FEP patients, n = 37; HCs, n=80) were included in the current study.
Data collection	All MR images were acquired with a Siemens 3T Trio scanner (Siemens, Erlangen, Germany) using a 12-channel head coil. For each participant, a high-resolution 3D T1-weighted image was obtained using a magnetization-prepared rapid gradient echo sequence (TR/echo time (TE) = 1670/1.89 ms, field of view (FOV) = 250 mm, flip angle (FA) = 9°, voxel size = 1.0×1.0×1.0 mm3, and sagittal slices = 208) for anatomical reference. Using an echo planar imaging sequence (TR/TE = 3500/30 ms, FOV = 240 mm, FA = 90°, voxel size = 1.9×1.9×3.5 mm3, 35 axial slices), rs-fMRI data were collected for 6 min and 58 seconds. The phase-encoding direction for all images was anterior to posterior. During rest, participants were instructed to keep their eyes closed and relax but to not fall asleep. To ensure that participants had not fallen asleep, they completed a questionnaire after the scan.
Timing	Data were collected from June 2010 to August 2016.
Data exclusions	From 40 FEP and 110 HC participants, we only included participants with ToM task scores (excluding 2 FEP patients and 21 HCs) and structural and functional MRI data with intact cerebella (excluding 1 FEP patient and 9 HCs). In total, 117 participants (FEP patients, n = 37; HCs, n=80) were included in the current study. Exclusion criteria were pre-established.
Non-participation	No participants dropped out.
Randomization	Allocation was not random, but participants were age-, sex-, and handed-matched.

Reporting for specific materials, systems and methods

We require information from authors about some types of materials, experimental systems and methods used in many studies. Here, indicate whether each material, system or method listed is relevant to your study. If you are not sure if a list item applies to your research, read the appropriate section before selecting a response.

Materials & experimental systems

n/a	Involved in the study	
☒	☐	Antibodies
☒	☐	Eukaryotic cell lines
☒	☐	Palaeontology and archaeology
☒	☐	Animals and other organisms
☐	☒	Human research participants
☒	☐	Clinical data
☒	☐	Dual use research of concern

Methods

n/a	Involved in the study	
☒	☐	ChIP-seq
☒	☐	Flow cytometry
☐	☒	MRI-based neuroimaging

Human research participants

Policy information about studies involving human research participants

Population characteristics	Forty first-episode psychosis (FEP) patients were recruited from the Seoul Youth Clinic of Seoul National University Hospital (SNUH). Using the Structured Clinical Interview for DSM, fourth edition (DSM-IV), Axis I (SCID-I), experienced psychiatrists interviewed all FEP individuals who met the following inclusion criteria: between the age of 15 to 40; diagnosis of schizophrenia, schizoaffective disorder, or schizophreniform disorder according to the DSM-IV criteria; and symptom presence for less than 2 years. A total of 110 healthy controls (HCs) were recruited from internet advertisements, screened, and confirmed using the SCID Nonpatient Edition (SCID-NP). Mean ages were 23.05 (5.64) for FEP group and 22.99 (4.76) for HC group. FEP group included 16 males and 21 females. HC group included 48 males and 32 females.
Recruitment	Forty first-episode psychosis (FEP) patients were recruited from the Seoul Youth Clinic of Seoul National University Hospital (SNUH). A total of 110 healthy controls (HCs) were recruited from internet advertisements, screened, and confirmed using the SCID Nonpatient Edition (SCID-NP).
Ethics oversight	The study was conducted in accordance with the Declaration of Helsinki and was approved by the IRB of the SNUH (IRB no. H-2104–223–1216).

Note that full information on the approval of the study protocol must also be provided in the manuscript.

Magnetic resonance imaging

Experimental design

Design type	resting state.
Design specifications	rs-fMRI data were collected for 6 min and 58 seconds.
Behavioral performance measures	N/A.

Acquisition

Imaging type(s)	functional
Field strength	3T
Sequence & imaging parameters	TR/TE = 3500/30 ms, FOV = 240 mm, FA = 90°, voxel size = 1.9×1.9×3.5 mm3, 35 axial slices
Area of acquisition	Whole brain scan was used.

Diffusion MRI ☐ Used ☒ Not used

Preprocessing

Preprocessing software	Image preprocessing was performed via the CONN toolbox (version 19c) as implemented in MATLAB 2020b (http://www.nitrc.org/projects/conn).
Normalization	linear normalization.
Normalization template	MNI space.
Noise and artifact removal	ARtifact detection Tools (ART)-based outlier detection was performed. The signals from white matter, cerebrospinal fluid, motion realignment parameters, and their first derivatives were regressed out (aCompCor strategy).
Volume censoring	N/A.

Statistical modeling & inference

Model type and settings	general linear model
Effect(s) tested	The coefficients of Pearson’s bivariate correlation were subsequently converted into normally distributed z scores via Fisher r-to-z transformation.

Specify type of analysis: ☐ Whole brain ☐ ROI-based ☒ Both

Anatomical location(s)	To study cerebellar FC with socially relevant cerebral regions, five social cerebellar ROIs were chosen according to the MNI coordinates revealed by the automated meta-analysis of NeuroSynth with the keywords “action” and “mirror” for the MNS seeds and “mentalizing” for the MENT seeds8. Spherical ROIs (5 mm) were created, centered around these coordinates. The centers of the two MENT cerebellar ROIs were located at the right crus II (+26, -84, -32) and its left mirror location (-26, -84, -32), while those of the three MNS cerebellar ROIs were at the right crus I (+40, -48, -32) and lobule VIII (+15, -75, -50),

(+25, -60, -50).

Statistic type for inference<br>(See Eklund et al. 2016)	We reported all relevant parameters for cluster-wise methods. cluster threshold: p-FDR corrected < 0.05; voxel threshold: p-uncorrected < 0.001.
Correction	As there were five cerebellar seeds, the seed-level Bonferroni procedure was used to account for multiple comparisons (Bonferroni corrected p < 0.01 (0.05/5)).

Models & analysis

n/a	Involved in the study
☐	☒ Functional and/or effective connectivity
☒	☐ Graph analysis
☒	☐ Multivariate modeling or predictive analysis

Functional and/or effective connectivity	Pearson correlation.
